# Cardiac Autonomic Neuropathy Is Not Associated with Apolipoprotein E Gene Isoforms in the Kazakh Population: A Case–Control Study

**DOI:** 10.3390/diagnostics14171978

**Published:** 2024-09-06

**Authors:** Nazira Bekenova, Alisher Aitkaliyev, Tamara Vochshenkova, Balzhan Kassiyeva, Valeriy Benberin

**Affiliations:** 1 Department of Science, Medical Centre Hospital of President’s Affairs Administration of the Republic of Kazakhstan, Astana 010000, Kazakhstan; aitkaliyev1998@gmail.com (A.A.); vochshenkova@gmail.com (T.V.); kassiyevabs@gmail.com (B.K.); valery-benberin@mail.ru (V.B.); 2 Institute of Innovative and Preventive Medicine, Astana 010000, Kazakhstan

**Keywords:** cardiac autonomic neuropathy, *APOE* gene, ε4 isoform, Kazakh population

## Abstract

The absence of an early diagnosis of cardiac autonomic neuropathy might increase the risk of the disease, progressing to an irreversible stage. Therefore, this study aims to investigate the *APOE* gene isoforms in patients with cardiac autonomic neuropathy to identify early markers for predicting this disease in the Kazakh population. A total of 147 patients with cardiac neuropathy and 153 controls were examined in this case–control study. Patients were genotyped for two polymorphisms of the *APOE* gene using real-time PCR. Statistical calculations were performed using binary logistic regression. As a result of our study, we found that there was no statistically significant difference in the frequency of any *APOE* gene isoforms (*APOE* (ε2/ε2), *APOE* (ε2/ε3), *APOE* (ε2/ε4), *APOE* (ε3/ε3), or *APOE* (ε4/ε4)) between the patient group and the control group (*p* = 0.69, *p* = 0.64, *p* = 0.19, *p* = 0.22, *p* = 0.97, respectively). Thus, cardiac autonomic neuropathy is not associated with *APOE* gene isoforms in the Kazakh population.

## 1. Background

Cardiac autonomic neuropathy (CAN) is a common but underdiagnosed complication of diabetes mellitus. It is closely related to and significantly impacts heart diseases such as ischemia, myocardial infarction, hypertension, orthostatic hypotension, heart failure, and arrhythmias [1]. Tissue damage may cause irregularities in heart rate and blood pressure, potentially increasing the risk of cardiovascular events. CAN is a result of microvascular complications linked to long-term type 2 diabetes, though it can also emerge before a formal diagnosis. CAN may manifest in various ways, including resting tachycardia, orthostatic hypotension, exercise intolerance, intraoperative cardiovascular instability, silent myocardial infarction, and a higher risk of mortality [2]. Risk factors for the development of CAN include diabetes mellitus, amyloidosis, porphyria, hypothyroidism, and cancer.

Additionally, hypertension, dyslipidemia, obesity, age, sex, body mass index (BMI), cigarette smoking, and alcoholism can also contribute to impaired cardiac autonomic function [2,3]. The pathogenesis of CAN is complex and poorly understood. Treatment generally focuses on the symptomatic control of orthostatic hypotension, which is a late complication, and current strategies for reversing the disease are limited [3].

According to the literature, the factors associated with the development of CAN include age, lipid metabolism disorders, other components of metabolic syndrome, arterial hypertension, and glucose metabolism disturbances [4]. Hyperglycemia and fatty acid metabolism play a crucial role in the development and progression of diabetic microvascular complications, including neuropathy [5]. In diabetes, microvascular disease mechanisms involve several pathological processes: the accumulation of advanced glycation end products (AGEs); the excessive production of endothelial growth factors; and abnormal activation of pathways such as protein kinase C (PKC), the polyol pathway, and the renin–angiotensin system (RAS) [6]. 

At the same time, there is some evidence that parasympathetic dysfunction can occur with minimal fluctuations in blood glucose levels and in the absence of insulin resistance or obesity [7]. Damage to the vagus nerve, the longest parasympathetic nerve that regulates about 75% of parasympathetic activity, can result in a reduced parasympathetic tone and resting tachycardia. Early autonomic dysfunction may not present with obvious symptoms and can only be identified through abnormal heart rate variability (HRV) indices [2]. 

As CAN progresses, it can disrupt the cardiovascular system, causing symptoms such as dizziness, palpitations, and lightheadedness. In advanced stages, both the parasympathetic and sympathetic nervous systems can be affected, potentially leading to the denervation of the cardiac nerves. This can result in a lack of response to exercise, stress, or sleep, posing significant risks [2,3]. 

The absence of clinical manifestations in the early stages and underdiagnosis can lead to the disease progressing to an irreversible stage. Therefore, using genetic variants associated with CAN may help identify individuals who are at high risk of developing the condition.

Apolipoprotein E (*APOE*) is a protein component of various lipoproteins, including very-low-density lipoproteins (VLDLs), intermediate-density lipoproteins, chylomicrons, and high-density lipoproteins (HDLs). It serves as a key ligand for three cell surface receptors: the low-density lipoprotein (LDL) receptor, the low-density lipoprotein-related protein (LRP), and the VLDL receptor. *APOE* mediates the catabolism of these lipoproteins by binding to the receptors in the liver [8]. 

*APOE* is one of the key proteins in lipoprotein and cholesterol metabolism [9]. The *APOE* gene has three isoforms: epsilon 2, epsilon 3, and epsilon 4 code for the isoforms ε2 (rs7412-T, rs429358-T), ε3 (rs7412-C, rs429358-T), and ε4 (rs7412-C, rs429358-C), respectively [10].

The presence of the *APO*Ε2 isoform on triglyceride-rich particles impairs their binding to lipoprotein receptors such as LDL receptors (LDLR), LRP, and heparan sulphate proteoglycans (HSPGs). This binding impairment leads to the defective clearance of chylomicrons and remnants of VLDL from the bloodstream, ultimately resulting in premature atherosclerosis [8].

Carrying the ε2/ε2 isoform, along with having diabetes, hypothyroidism, obesity, and other factors, pose a risk of developing dysbetalipoproteinemia, which was previously known as WHO hyperlipoproteinemia type III or Frederickson Classification type III. Once patients develop type III hyperlipoproteinemia or dysbetalipoproteinemia, they are at an increased risk of developing atherosclerotic cardiovascular disease [11]. *APOΕ* (ε4/ε4) is a risk factor for susceptibility to coronary heart disease (CHD) and Alzheimer’s disease [12,13,14]. *APO*Ε3 is the most common isoform across all populations and is considered the wild-type variant [15]. 

Currently, there are only a limited number of studies focusing on the significance of the *APOE* gene in diabetic neuropathy. Chang Gao et al. found that the frequency of the ε2/3 genotype was higher in the group with diabetic neuropathy (19.3% compared to 9.1%, *p* = 0.01) compared to those without diabetic neuropathy [16]. However, this study focused on general diabetic neuropathy. Regarding the study of genetic factors in CAN, there are many works examining the role of other SNPs in different genes. In our previous research, we investigated the role of SNPs in genes such as SNCA, FTO, PPARG, FLACC1, and XRCC1 [17].

This study aims to investigate the significance of *APOE* gene isoforms in CAN regardless of the presence of diabetes to identify the risk factors for this condition among individuals of Kazakh nationality.

## 2. Materials and Methods

### 2.1. Study Design and Patient Selection

This is a case–control study, which included 147 patients with CAN and 153 patients without CAN, regardless of diabetes presence. All study participants were of Kazakh nationality. Patient recruitment was conducted in the therapeutic department of the Medical Center Hospital of the President’s Affairs Administration of the Republic of Kazakhstan (the Hospital) from September 2017 to August 2020.

Cases were continuously selected from patients in the therapeutic department of the Medical Center Hospital of the President’s Affairs Administration of the Republic of Kazakhstan from September 2017 to August 2020, with the hospital’s turnover being 25,454 patients at the start of the study in 2017. The control group consisted of individuals who underwent preventive examinations at the same hospital and were confirmed to be free of CAN.

Demographic data—including gender, age, height, weight, and ethnicity—were obtained from the medical records of the study participants. All personal patient data were encoded to avoid bias and maintain confidentiality. Levels of glucose, glycated hemoglobin, cholesterol, triglycerides, HDLs, and LDLs were determined using standard methods by venous blood collection after fasting.

To diagnose CAN, Holter monitoring was used to obtain instrumental data. The 24 h Holter monitoring was conducted with the Medilog DARWIN ECG monitoring system from Switzerland. 

The following parameters were evaluated:

- Average Standard Deviation of NN Intervals (SDNN av) (ref. interval 53–279 m/s);
- Median Standard Deviation of NN Intervals (SDNN med) (ref. interval > 53.8 m/s);
- Average Standard Deviation of Average NN Intervals (SDANN av) (ref. interval 45–261 m/s);
- Average Root Mean Square of Successive Differences (RMSSDs av) (ref. interval 7–103 m/s);
- Median Root Mean Square of Successive differences (RMSSDs med) (ref. interval 28.8–71.9) m/s);
- Average Percentage of NN Intervals differing by more than 50 ms (pNN50 av) (ref. interval 0–137%);
- Median Percentage of NN Intervals differing by more than 50 ms (pNN50 med) (ref. interval 6.0–44.1%);
- High-Frequency Power (HF) (>56.4);
- Low-Frequency Power (LF) (<43.6);
- Ratio of High-Frequency Power to Low-Frequency Power (HF/LF) (<0.8).

The above values were used as normal values to select patients for the control group, and deviations from these values were used for the diagnosis of CAN and selection for the case group. CAN was characterized as deviations of three or more HRV measurements (such as SDNN, RMSSD, HF, LF, and HF/LF) [18,19]. 

Hypertension was determined by observing an elevation in systolic blood pressure (SBP) equal to or greater than 140 mm Hg and/or diastolic blood pressure (DBP) equal to or greater than 90 mm Hg, either through the daily monitoring of blood pressure or by assessing the consistent use of antihypertensive medication. The BTL-08 ABPM ambulatory blood pressure recorder (BTL Industries Ltd.), manufactured in Newcastle, UK, was used for the daily monitoring of blood pressure.

The diagnosis of type 2 diabetes (T2DM) was made based on the clinical protocol established by the Ministry of Health of the Republic of Kazakhstan. The diagnosis of diabetes mellitus was based on a fasting glucose level of ≥7 mmol/L and a hemoglobin A1c (HbA1c) level of ≥6.1% (48 mmol/mol).

Blood samples were taken from the cubital vein in the procedural room after a 12 h fast. Plasma was separated by centrifugation at 1000× *g* (4 °C) for 10 min. Plasma, for further biochemical analysis, was stored at −30 °C. The serum obtained after centrifugation was used for analysis on the same day as blood collection. Glucose levels, total cholesterol, triglycerides, HDLs, and LDLs were measured using the enzymatic method on the Architect’s 8000 automated biochemical analyzers manufactured by Abbott Laboratories, Lake Forest, IL, USA. BMI was calculated by dividing weight in kilograms by the square of height in meters.

Inclusion criteria for the case group were an established diagnosis of CAN (regardless of diabetes presence), age 18 years and older, and Kazakh nationality. Exclusion criteria included genetic diseases in medical history, hypothyroidism or hyperthyroidism, rhythmic disturbances of cardiac electrical activity, left ventricular assist device (LVAD) placement within the last 3 months, regular alcohol consumption (more than 80 mg/day), anemia (Hb < 110), cancer, kidney disease, severe cardiovascular diseases, liver disease, a terminal stage of hematopoiesis, autoimmune diseases that may affect autonomic nerve fibers such as systemic lupus erythematosus, concomitant degenerative diseases (e.g., Parkinson’s disease or multiple system atrophy), medications that may affect heart rate (such as beta-blockers, verapamil, diltiazem, amiodarone, or nitrates), and pregnant and lactating women.

Inclusion criteria for the control group were the exclusion of CAN diagnosis, age 18 years and older, and Kazakh nationality. The exclusion criteria were similar to those in the case group.

### 2.2. Genotyping

All patients were genotyped for two polymorphisms, rs7412 and rs429358, of the *APOE* gene. The genotyping used advanced OpenArray technology, which facilitated reactions in small volumes. Custom-designed OpenArray slides (Thermo Fisher Scientific Inc., Waltham, MA, USA), each containing 3072 data points, were utilized. Pre-extracted DNA samples were combined with the reaction mixture in a 384-well sample plate for genotyping. Each sample necessitated 3.0 μL of an OppenArray real-time master mix (Thermo Fisher Scientific Inc.) and 2.0 μL of a DNA sample with a concentration of 50 ng/μL. The total volume per well was 5 μL, with each sample duplicated. After thorough mixing and centrifugation, probes were designed using the QuantStudio OpenArray AccuFill Plate Configurator (Thermo Fisher Scientific Inc.), and dried assays were placed in specific through-holes of the genotyping plates. These plates were specially engineered to accommodate two allele-specific probes, a minor groove binder, and two PCR primers, ensuring precise and accurate genotyping calls. The OpenArray technology utilizes nanoliter fluidics and can be tailored with up to 3072 through-holes in various configurations.

A plate-setup file was generated to delineate the protocol for the applied samples, incorporating analysis details. This file was then uploaded onto the QuantStudio™ 12K Flex software for experiment generation and execution. The prepared chips were inserted into the QuantStudio 12K Flex instrument (Thermo Fisher Scientific Inc.) using disposable genotyping blocks. Amplification reaction occurred through real-time PCR microfluidic technology. The resultant data from the amplification reaction were analyzed using online tools provided by the Thermo Fisher Cloud service. The bioinformatics analysis outcomes facilitated the categorization of the studied genes as homozygotes for the major allele, homozygotes for the minor allele, or heterozygotes.

### 2.3. Statistical Analysis

Quantitative data are presented as means (M + SD), medians, upper and lower quartiles, and Me (Q1, Q3) and used as continuous variables. The normality of data distribution was assessed using the Shapiro–Wilk test. A significance level of *p* < 0.05 was considered for determining statistically significant differences. Qualitative data were presented as frequencies and proportions. Variables were dichotomized as follows: gender (male/female) and presence of outcome or feature (yes/no).

Quantitative data were analyzed using the non-parametric Mann–Whitney test for independent groups. The frequency of the isoforms (*APOE* ε2, *APOE* ε3, *APOE* ε4, *APOE* (*e2*/*e2*), *APOE* (ε2/ε3), *APOE* (ε2/ε4), *APOE* (ε3/ε3), *APOE* (ε3/ε4), and *APOE* (ε4/ε4)) was compared between the case and control groups using the Chi-square test. Initial comparisons were made between 147 patients with CAN and 153 controls. Further analysis was performed using binary logistic regression to evaluate the impact of additional risk factors for CAN, including age, gender, total cholesterol, BMI, triglycerides, HDLs, LDLs, glucose levels, and the presence of diabetes. Data analysis was conducted using SPSS 26.0 statistical software.

### 2.4. Ethics

This research adhered to ethical principles and was approved by the hospital’s Local Commission on Bioethics, with permission note No. 5, issued on 27 September 2017. All medical procedures and tests were conducted under the approved standard operating procedures of the hospital. Before participation, all individuals willingly consented to be part of the study and provided informed consent by signing appropriate documentation.

## 3. Results

### 3.1. Clinical, Demographic, and Genetic Parameters of Patients

The average age of patients with CAN was slightly higher compared to those without the condition (53.8 ± 9.3 versus 53.4 ± 10.1, respectively). Among the CAN patients, males predominated. However, there were no statistically significant differences in age and gender between the two groups, as shown in Table 1.

Comparisons of glucose levels, triglycerides (TGs), total cholesterol, and LDL-C revealed no significant statistical differences between the groups. However, the average BMI was slightly higher in the CAN group compared to the non-CAN group. Conversely, HDL-C levels were lower in the CAN group, and this difference was statistically significant. Diabetes was more prevalent in the control group than in the CAN patients (72% versus 66%, respectively), but this difference was not statistically significant (Table 1).

In our sample, the most frequently observed *APOE* isoform was ε3/ε3. Its frequency was higher in the control group compared to the patients with CAN, but this difference was not statistically significant. The ε2/ε3 isoform was less common. While it was less frequent in the CAN group than in the control group, this difference was also not statistically significant. The rarest isoforms were ε4/ε2, ε4/ε4, and ε2/ε2. Comparing the frequencies of ε4/ε2 and ε2/ε2 isoforms between the case and control groups showed no significant differences in the number of carriers of these variants. Similarly, although the prevalence of the ε4/ε4 variant was slightly higher in the control group compared to the patients with CAN, this difference was not statistically significant either.

### 3.2. Relationship between the Clinical and Demographic Factors and CAN in Binary Logistic Regression

Age and gender did not influence the risk of CAN in any of the groups according to the logistic regression analysis, as illustrated in Table 2. Logistic regression analysis showed that factors such as BMI, glucose, triglycerides, total cholesterol, HDL-C, and LDL-C did not affect the development of CAN in our sample. Additionally, the presence of diabetes did not show a statistically significant effect on the risk of developing CAN.

## 4. Discussion

The risk of developing CAN is not associated with the *APOE* ε2/ε2, *APOE* ε2/ε3, *APOE* ε2/ε4, *APOE* ε3/ε3, *APOE* ε3/ε4, or *APOE* ε4/ε4 isoforms in the Kazakh population. Additionally, we found that patients with CAN had lower HDL-C levels and increased BMI.

The role of *APOE* in CAN is currently only indirectly understood. However, research highlights its importance in cardiovascular damage mechanisms related to hyperglycemia. For instance, studies have shown that in *APOE* knockout mice, the increased expression of human aldose reductase accelerates atherosclerosis [20]. In mice with null *APOE* and a specific overexpression of PKCβ2 in endothelial cells, the severity of atherosclerotic lesions increases by 70% [21]. 

In addition to vascular damage in the context of insulin resistance and hyperglycemia, CAN involves the impaired innervation of cardiac muscle. Metabolic disturbances, such as the activation of the polyol pathway for glucose utilization, non-enzymatic glycosylation of proteins, disruptions in the cyclooxygenase cycle, and excessive free radical formation, contribute to neuronal demyelination and reduced nerve fiber conductivity [22]. There is also evidence suggesting that *APOE* isoforms may influence myelination processes. For example, humanized mice with the *APOE* ε4 isoform showed less myelination compared to those with the *APOE* ε3 isoform [23]. Although these studies were conducted on tauopathy mouse models, they suggest the potential role of *APOE* isoforms in disease pathogenesis. Additionally, it has been reported that carrying at least one *APOE* ε4 allele is associated with lower levels of *APOE* in plasma [24].

Isoform pairs in the *APOE* gene are often considered markers for the late development of Alzheimer’s disease. Additionally, the ε4 isoform (rs429358-C allele, rs7412-C allele) has been associated with the risk of developing CHD (*p* = 2.7 × 10^−18^) [25,26]. Alharbi K. et al. identified a strong association between the *APOE* gene and diabetes, where the ε4 isoform of the *APOE* gene increased the chances of developing diabetes by nearly 4.5 times [27]. Moreover, allelic variations in the *APOE* gene (ε2 and ε4), which influence the lipid profile, may contribute to the risk of complications in diabetic patients [27]. However, in our study, we did not find an association with any of the *APOE* gene isoforms. 

*APOE* is a lipid transport protein that binds to various types of lipids, including cholesterol, phospholipids, and triglycerides (TGs), in lipoprotein particles [28]. *APOE* facilitates lipid transport into cells through different cell-surface receptors, with the transport mechanism via LDLR being the most extensively studied [29,30]. Although the role of *APOE* in the physiology of HDL is less well understood, there is evidence that *APOE* plays a significant role in HDL metabolism, particularly through interactions with lecithin cholesterol acyltransferase (LCAT). *APOE* has been reported to activate LCAT in vitro. Additionally, the presence of a specific apolipoprotein or a related factor is known to influence LCAT activity. Studies in mice with the double knockout of APOA and *APOE* have suggested that *APOE* is a potential activator of LCAT [31].

LCAT is an enzyme that converts free cholesterol in HDL into cholesterol esters. This process is crucial for “reverse cholesterol transport”, which is necessary for removing cholesterol from the peripheral tissues [32]. However, *APOE* isoforms affect cholesterol esterification differently. For instance, reconstituted HDLs (rHDLs) containing *APOE* ε2 are associated with a higher maximum rate of cholesterol esterification compared to the particles containing *APOE* ε3 and *APOE* ε4 [33].

Our study found that patients with CAN had lower HDL-C levels compared to the control group. According to Q Liu et al., carriers of the *APOE* ε4 isoform had lower HDL-C levels than those with ε3 or ε2 isoforms [34]. However, we did not investigate the impact of *APOE* gene isoforms on lipid levels. Further research examining the effect of the *APOE* gene on HDL-C with CAN may be valuable.

Among the limitations of our study, the small sample size stands out as the primary point. A larger sample size might have allowed us to identify the genetic factor linkage with CAN in patients with diabetes. Secondly, we did not assess the influence of *APOE* gene polymorphisms on the level of lipid metabolism indicators. However, this does not fully explain the patterns of CAN development in our sample. Additionally, determining HDL-C levels alone is insufficient to assess functional abnormalities in HDL metabolism. Studying the impact of the gene isoforms on protein production can expand our understanding of the *APOE* gene’s association with the development of CAN. Additionally, the patient set was recruited within only one hospital, which did not allow us to extrapolate our results to the entire population, that is, to the entire Kazakh population.

Among the advantages of our study, it is important to highlight that we made the first attempt to investigate the baseline *APOE* isoform in relation to CAN among individuals of Kazakh ethnicity, which revealed no association with the disease.

## 5. Conclusions

Despite CAN being considered a complication of diabetes, in our study, it was diagnosed even in patients without diabetes. However, we cannot assert that these patients have no risk of developing diabetes. We can only speculate that CAN may develop long before the diagnosis of diabetes. In our study, CAN was not associated with genetic factors; however, the phenotype of reduced HDL-C levels may be one of the risk factors for the disease in this population. Additionally, we found that patients with CAN had lower HDL-C levels. Given these findings, reduced HDL-C concentrations and impaired LCAT function could contribute to an increased risk of atherosclerosis, potentially leading to cardiovascular complications.

These findings could guide targeted studies of these mechanisms in future research, which may contribute to the development of preventive recommendations.

## Figures and Tables

**Table 1:** Clinical and demographic parameters of patients.

	Patients with CAN (*n* = 147)	Patients without CAN (*n* = 153)	*p*-Value
Age (M + SD)	53.8 ± 9.3	53.4 ± 10.1	0.44 ^a^
Gender (absolute value, %)			
male	88 (59.9%)	89 (58.2%)	0.77 ^b^
female	59 (40.1%)	64 (41.8%)	
BMI Me (Q1, Q3)	30.03 (27.1; 30.9)	29.3 (27.0; 32.0)	0.03 ^a^
Blood glucose (mmol/L)	6.21 (5.3; 9.4)	5.96 (5.1;8.2)	0.51 ^a^
Triglyceride (mmol/L)	1.66 (1.18; 2.97)	1.73 (1.12; 2.94)	0.83 ^a^
T2DM presence (abs, %)			
Yes	66 (44.9%)	72 (47.1%)	0.07 ^b^
No	81 (55.1%)	81 (52.9%)	
Total cholesterol	5.5 (4.6; 6.1)	5.4 (4.8; 6.2)	0.95 ^a^
Low-density lipoprotein (LDL-C)	3.2 (2.8; 4.0)	3.4 (2.7; 3.9)	0.87 ^a^
High-density lipoprotein (HDL-C)	1.1 (0.9; 1.3)	1.2 (1.0; 1.5)	0.04 ^a^
*APOE*			
ε2	13	19	0.32 ^b^
ε3	101	114	0.17
ε4	33	20	0.03
ε2/ε2	2	3	0.69
ε2/ε3	13	16	0.64
ε2/ε4	1	4	0.19
ε3/ε3	99	113	0.22
ε4/ε4	3	3	0.97
ε4/ε3	0	0	- ^c^

^a^—The Mann–Whitney U test was used to compare mean values. ^b^—Comparisons were made using the Chi-square test. ^c^—Comparisons were not conducted. M—Mean; SD—Standard Deviation; M—Median; T2DM—Type 2 diabetes mellitus

**Table 2:** Relationship between the clinical and demographic factors and CAN in binary logistic regression.

	OR (95% CI)	*p*-Value
Age	1.01 (0.99–1.05)	0.18
Gender	0.99 (0.60–1.65)	0.98
BMI, kg/m^2^	1.05 (0.99–1.11)	0.07
Glucose (mmol/L)	0.98 (0.91–1.06)	0.61
TG (mmol/L)	1.09 (0.93–1.27)	0.29
Total cholesterol	0.95 (0.70–1.29)	0.73
Low-density lipoprotein (LDL)	1.03 (0.73–1.46)	0.86
High-density lipoprotein (HDL)	0.50 (0.22–1.17)	0.11
T2DM presence	1.33 (0.74–2.42)	0.34

TG—triglyceride; T2DM—Type 2 diabetes mellitus.

## Data Availability

The data presented in this study are available upon request from the corresponding author due to the necessary protection of primary data.

## Abbreviations

CAN	Cardiac autonomic neuropathy
OR	Odds ratio
BMI	Body mass index
AGEs	Advanced glycation end products
PKC	Protein kinase C
RAS	Renin–angiotensin system
HRV	Heart rate variability
*APOE*	Apolipoprotein E
VLDL	Very-low-density lipoproteins
HDL	High-density lipoproteins
LDL	Low-density lipoprotein
LRP	Lipoprotein-related protein
LDLR	LDL receptors
HSPG	Heparan sulphate proteoglycans
CHD	Coronary heart disease
ECG	Electrocardiogram
SDNN av	Average Standard Deviation of NN Intervals
SDNN med	Median Standard Deviation of NN Intervals
SDANN av	Average Standard Deviation of Average NN Intervals
RMSSD av	Average Root Mean Square of Successive Differences
RMSSD med	Median of Root Mean Square of Successive Differences
pNN50 av	Average Percentage of NN Intervals differing by more than 50 ms
pNN50 med	Median Percentage of NN Intervals differing by more than 50 ms
HF	High-Frequency Power
LF	Low-Frequency Power
HF/LF	The Ratio of High-Frequency Power to Low-Frequency Power
SBP	Systolic blood pressure
DBP	Diastolic blood pressure
T2DM	Type 2 diabetes
LVAD	Left ventricular assist device
Hb	Hemoglobin
TG	Triglycerides
IDL	Intermediate-density lipoproteins
LCAT	Lecithin cholesterol acyltransferase
rHDLs	Reconstituted HDLs
